# Clinical performance of class II MOD fiber reinforced resin composite restorations: an 18-month randomized controlled clinical trial

**DOI:** 10.1186/s12903-025-05521-5

**Published:** 2025-01-30

**Authors:** Mohamed Hamdy Mohamed, Eman Ali Abouauf, Rania Sayed Mosallam

**Affiliations:** 1 Conservative Dentistry Department, Faculty of Dentistry, Cairo University, Cairo, Egypt; 2 Conservative Dentistry Department, Faculty of Oral and Dental Medicine, Ahram Canadian University, Giza, Egypt

**Keywords:** Short fiber, Ever-X posterior, Ribbond, Polyethylene fiber, Clinical performance

## Abstract

**Background:**

In recent years, there have been suggestions for new restorative strategies that aim to effectively utilize modern adhesive technologies and protect the remaining intact tooth structure. This study was conducted to evaluate the clinical performance of fiber reinforced resin composites in restoring Class II MOD cavities over 18 months.

**Methods:**

Forty-five participants with class II MOD cavities were randomly enrolled. The participants were allocated to three groups (*n* = 15): Group (1) short glass fiber reinforced resin composite (EverX Posterior), Group (2) polyethylene fiber (Ribbond-Ultra) reinforced resin composite and Group (3) only conventional resin composite (Filtek Z250). The modified USPHS criteria were used to evaluate the restorations at baseline; and at three, six, 12 and 18 months. The categorical data were reported as frequencies and percentages. Intergroup comparisons were analyzed using Chi-Square test after Bonferroni correction (*P* ≤ 0.016). Comparisons within each group were analyzed using Cochran’s Q test after Bonferroni correction (*P* ≤ 0.005).

**Results:**

Intergroup comparisons revealed no statistically significant differences in any of the evaluated criteria except for color match. Compared with the other groups, the short glass fiber-reinforced restoration group presented significantly fewer color matches at different follow-up periods.

**Conclusions:**

Class II direct fiber-reinforced resin composite restorations can offer acceptable performance similar to the nanohybrid resin composite through 18-month of clinical service.

**Clinical significance:**

Fiber reinforcement of resin composites could be a reliable technique for restoring posterior cavities considering factors such as application complexity, cost and aesthetic considerations.

**Trial registration:**

The trial was registered retrospectively on (19/05/2022) at https://clinicaltrials.gov with the ID (NCT05380973).

## Introduction

Contemporary restorative dentistry necessitates the establishment of a mechanically, and adhesively integrated interface between the restoration and the tooth, capable of withstanding repetitive functional stresses over an extended duration [1]. This approach involves replacing the missing tooth structure with restorative materials that have biomechanical characteristics similar to those of the sound tooth structure.

The loss of one or two marginal ridges results in a significant reduction in tooth stiffness as a result of extending the cavity preparation to class II or a mesio-occluso-distal (MOD) [2]. It has been reported that the stresses created in the remaining tooth structure increases with restoration volume [3] and the residual cusp acts under occlusal force as a cantilever beam. Cusp bending uses the cavity’s floor as a fulcrum, and as the cavity’s depth increases, the cantilever length increases resulting in more cuspal deflection [4].

The use of fiber reinforcement in resin composite restorations has been suggested to increase the fracture toughness in cavities subjected to high levels of stresses, as these restorations have shown favourable results in many previous studies [5–9]. Fibers have demonstrated the ability to regulate the stresses caused by polymerization shrinkage through fiber orientation. Thus, there has been a reduction in marginal microleakage when compared with traditional resin composites [10]. The fibers in the material serve the purpose of enhancing its structural qualities by acting as barriers to cracks. As a result, the fiber structure serves to augment the mechanical characteristics of composite materials making them suitable for direct restorations of extensive cavities in vital and endodontically treated teeth [10]. Furthermore, a systematic review of laboratory studies reported in 2021 that fiber reinforcement of resin composite restorations resulted in increased fracture resistance and marginal adaptation when compared to the conventional ones [11].

EverX Posterior (GC Corporation, Japan) is a fiber reinforced resin composite that contains randomly oriented discontinuous short electric (E)-glass fibers. Compared with conventional resin composites, the use of short fibers increased the mechanical properties of resin composite restorations [12]. When used as a dentin substitute in extensive direct proximal restorations, it showed a comparable performance to that of indirect ceramic restorations offering a cost-effective technique for extensive restorations [13, 14].

Ribbond Ultra (Ribbond Inc., Seattle WA USA) fibers are long continuous ultra-high-molecular-weight polyethylene fibers; these fibers have a cross-linked leno weave design, characterized by locked nodal intersections [15]. In addition, the fibers are oriented in multiple directions, allowing the energy from the forces to absorbed and distributed favourably, thereby decreasing stress values [16]. Furthermore, polyethylene fibers have been reported to increase fracture resistance of MOD resin composite restorations [3] and marginal adaptation by protecting the adhesive interface from polymerization stresses in deep cavities [17].

Owing to the scarcity of evidence-based literature regarding the clinical application of the fiber reinforcement approach in different clinical scenarios, it was advantageous to clinically assess fiber reinforced resin composites in class II MOD restorations through a randomized clinical trial. The class II MOD cavity configuration is one of the most challenging scenarios among cavity designs in terms of the longevity of the restorations. Therefore, this trial was conducted to evaluate the clinical performance of class II MOD fiber reinforced resin composite restorations. The tested null hypothesis was that fiber reinforced resin composites would exhibit similar clinical performance to nanohybrid resin composite in class II MOD restorations over an 18-month follow-up period.

## Materials and methods

### Study setting

The procedures conducted in this trial adhered to the ethical guidelines reported by the Research Ethics Committee of Cairo University (CREC) (approval number: 16-06-2022). The trial was reported adhering to the guidelines set by CONSORT. The participants were enrolled from the educational clinic of the faculty of dentistry, Cairo University, Egypt. Comprehensive information was provided to all participants describing the study’s objectives, methods, safety measures, advantages, and anticipated duration of participation. Accordingly, informed consent to participate was obtained from all the participants in the study before the trial commenced. The research proposal was registered at (www.clinicaltrials.gov) I.D: NCT05380973.

### Trial design

The study was an 18-month randomized clinical trial with a double-blinded parallel design and three arms, an equivalence trial with the same allocation ratio.

### Sample size calculation

A power analysis was performed to statistically test the research null hypothesis. According to the results of Ernst et al. [18], the outcome was marginal adaptation using modified USPHS. The probability of score A for the marginal adaptation of the nanohybrid resin composite (comparator) was (0.96), and the probability of score B was (0.04), with an effect size of w = 0.92 (*n* = 10). The estimated probability of marginal adaptation of the short fiber reinforced resin composite (intervention 1) was (0.9) for score A, and (0.1) for score B with an effect size of w = 0.8 (*n* = 13), and the estimated probability of marginal adaptation for the polyethylene ribbon fiber reinforced resin composite (intervention 2) was (0.9) for score A, (0.1) for score B with an effect size of w = 0.8 (*n* = 13).The estimated sample size (n) was (36) restorations which was determined to be adequate to have power of 80%, while the alpha (α) level was 0.05 (5%). The sample size was increased to (45) restorations (*n* = 15) for each group to compensate for possible participants quitting during the study duration.

### Eligibility criteria

The study included the participants who met the criteria shown in Table 1.

**Table 1:** Inclusion and exclusion criteria

Inclusion criteria	Exclusion criteria
1. Males and females aged 20–50 yrs. 2. Good oral hygiene 3. Cooperative patients who agreed to participate in the trial 4. Class II MOD carious lesions in vital posterior teeth. 5. Class II MOD caries of ICDAS scores 3 or 4,5 not involving more than 2/3 dentin thickness (confirmed radiographically) 6. Healthy periodontal status 7. The teeth present in normal occlusion and contact with adjacent teeth.	1. Systematic disease 2. Pregnancy 3. Evidence of parafunctional habits 4. Allergy to any of the materials used in the trial 5. Hypersensitivity 6. Secondary caries 7. Prolonged pulpal response following sensibility tests 8. Negative pulpal response or periapical radiolucency indicating necrosis and/or periapical pathosis.

### Randomization, allocation and blinding

Simple randomization for teeth was performed by generating numbers ranging from 1:45, according to the principles of randomized trials, Each generated number was randomly allocated to either the interventions or control group, an a randomization list was generated using a computer (www.randomization.com). Patients were allocated according to the order of their order of presentation to the clinic, where they were examined following the eligibility criteria. For every participant, only one material was used. Each participant was given a sequentially numbered opaque sealed envelope that included the previously generated treatment group code. The envelopes were organized by a third party, ensuring that the randomization process was not influenced by the principal investigators or assessors. Both the patients and the examiners were blinded to the material allocation. Therefore, the outcome examiners were not included in the preliminary examination. Despite the envelope being opened following cavity preparation, the operator was not fully blinded because of the variation in packaging and manipulation of the restorative materials.

### Restorative procedures

All cavity preparation steps and restorative procedures were performed by the principal investigator; shade selection was performed before cavity preparation using vita classical shade guide. Teeth were isolated with heavy consistency rubber dam sheets in multiple isolation sequences to ensure a lack of contamination of the operative field [19]. Class II MOD cavities were prepared in accordance with the concepts of minimally invasive dentistry [20] by removing only carious tissues and creating peripheral zone of clean sound enamel, DEJ and 1–2 mm dentin. Access to the proximal carious lesions was gained using high-speed spherical carbide bur (MANI, INC, Japan) with a copious water coolant. Carious dentin was removed selectively to obtain firm dentin using a low speed round carbide bur (MANI, INC, Japan) and a sharp excavator (Zeffiro, Lascod, Italy) according to recent caries removal clinical guidelines [21]. Tactile manipulation with an excavator was performed to ensure firm dentin was reached (dentin does not deform and resistant to manual excavation, but can be removed with some pressure). Each bur was replaced after five cavity preparations [22]. The finishing process of cavity margins was performed using yellow coded finishing diamond stones (#368EF, Komet, USA).

To restore the missing proximal walls [23], an appropriate precontoured sectional matrix (TOR VM Dental Manufacturing Company, Russia) and a properly sized plastic diamond wedge (Bioclear Matrix Systems, Tacoma, USA) were applied and stabilized using a separation ring (Composi-Tight^®^ 3D Fusion™ Matrix Ring, Garrison^®^ Dental Solution, USA). The selective enamel etching technique using 37% phosphoric acid gel was applied for only 15 s [24]. A universal adhesive was applied to the etched enamel and dentin surfaces according to the manufacturer’s instructions [25] and then light cured for 20 s using a light emitting diode (LED) light curing device with a light intensity of up to 1600 mW/cm2 (Curing Pen, Eighteeth, Changzhou Medical Technology Co.Ltd). The light output of the curing unit was monitored and rechecked every 10 uses with a curing light intensity meter (Woodpecker, China). A flowable composite layer with a thickness of approximately 0.5 mm was applied to the cavity floor [3] using a gold plated composite applicator (Nordent Manufacturing Inc. Illinois, USA) and then was light-cured for 20s.

The proximal walls in both intervention and control groups were built using centripetal technique, which transformed the class II MOD cavity into a class I cavity [3]. A nanohybrid resin composite with a thickness of 1.5 mm was applied to build both the mesial and distal walls, then light cured from the occlusal direction for 20s.

Regarding EverX Posterior, a unitip of short fibers reinforced composite was applied using a unitip applicator (Cotisen unitips applicator) as reported by the manufacturer [26]. An increment of 2–3 mms was applied in a bulkfill technique to ensure that there is a minimum of 1–2 mm of occlusal space remained for the application of the overlaying nanohybrid resin composite. The fiber reinforced composite was light cured for 20s according to the manufacturer’s instructions, followed by application of occlusal layer of nanohybrid resin composite [27].

**Fig. 1:**
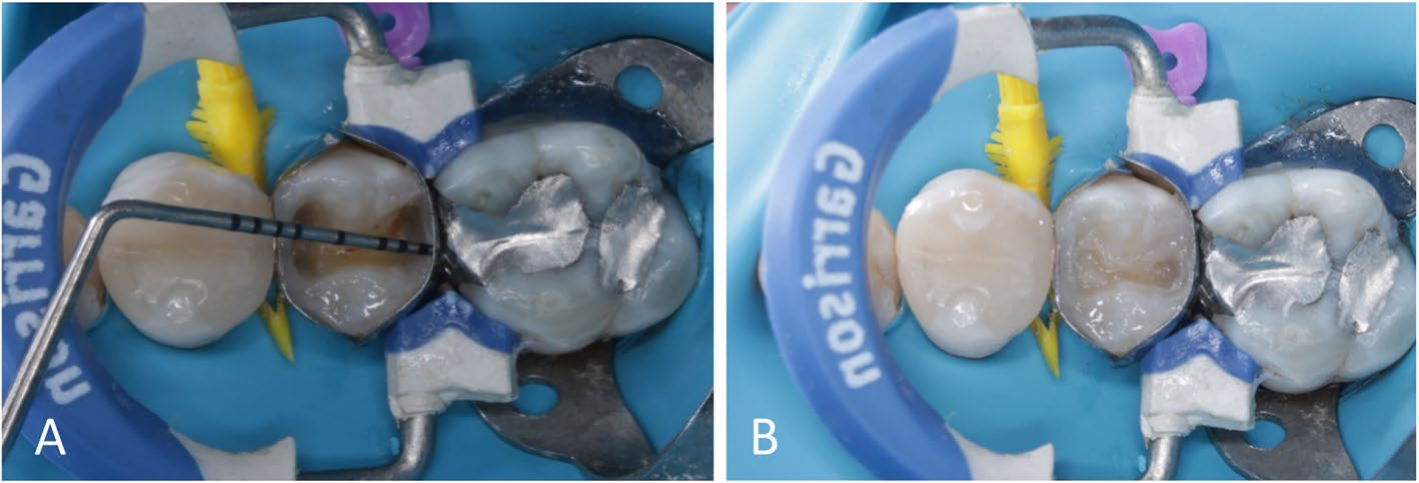
**A** Measuring the mesio-distal width, **B** Ribbond fibers were placed lining the floor and the cavity walls

Regarding polyethylene fibers, the mesio-distal distance and the bucco-lingual width of the cavity were measured using a graduated periodontal probe (Nordent Manufacturing Inc. USA) to select the correct length and width of the fibers to accurately conform into the cavity. Pieces of 2 mm wide polyethylene fiber were cut to the measured length with specific Ribbond scissors. After that, the polyethylene fibers were wetted with a resin wetting liquid for 2 min in a dark environment to avoid premature curing by soaking the fibers in the wetting liquid in a dispensing dish covered by a light filtering plate [28]. The first fiber was embedded into the layer of uncured flowable composite on the pulpal/gingival floor as close as possible to the tooth structure and oriented in a mesio-distal direction. The rest of fiber pieces were c-shaped using a ball burnisher to be easily adapted to cavity walls and placed circumferentially lining the facial, palatal and proximal walls stopping at an imaginary dentin‒enamel junction (DEJ) line [16]. The rest of the cavity was rebuilt with nanohybrid resin composite applied with an oblique incremental technique (Fig. 1). Each increment was light cured from the occlusal surface for 20 s using from the occlusal direction.

Regarding the control group, the proximal walls were built as described earlier, then the rest of the cavities were restored with a nanohybrid resin composite applied using an oblique incremental approach. The material was added in successive oblique increments. Each increment was light cured from the occlusal surface for 20 s using LED curing. All restorations were finished under using fine grit yellow coded tapered diamond stone (MANI, INC, Japan) attached to a high-speed contra angle hand-piece (Alegra TE-97 RM, W&H, Austria). Checking of any occlusal premature contact was performed through both patients’ sensibility and markings by articulating paper (Blue Red Combo 0.0028”/71 µm, Crosstex ^®^ International, USA). High spots were removed with yellow coded stones (MANI, INC, Japan). Polishing was done using composite polishing kits (DIACOMP PLUS TWIST, EVE, Germany), (KENDA^®^, Vaduz, Liechtenstein) and a felt wheel with aluminium oxide polishing paste. All the restorative materials were used according to the manufacturer’s guidelines (Table 2).

**Table 2:** Materials used in this study

Materials	Material description	Composition	Manufacturer	Lot no.
N etch	Acid etchant	Phosphoric acid (37%), thickening agent and color pigments	Ivoclar, Schaan, Liechtenstein	Z03XMT
Single Bond Universal Adhesive	Universal adhesive bonding agent	Dimethacrylate, MDP, HEMA, polyalkenoid acid copolymer, 5-nm silane-treated colloidal silica, ethanol, water, photoinitiator	3 M Oral Care Deutschland GmbH, Germany	00924B
EverX Posterior	A short glass fiber reinforced resin composite (SFRC)	**Resin matrix**: Bis-GMA, TEGDMA, PMMA. **Filler Content**: Silicon dioxide (max. 5 wt%), Barium glass (max. 70 wt%), Silanated E-glass fiber (max. 15 wt%). **Filler loading**:76 wt%, 57 vol%	GC Corporation, Japan	2,203,241
Ribbond-Ultra	Ultra-high molecular weight long continuous polyethylene fibers	Silanized polyethylene fibers	Ribbond Inc., Seattle, WA, USA	+D758U0/$$72C7/16D20221223/K
Ceramage modelling liquid	A resin wetting liquid	UDMA, Dimethyl Aminoethyl Methacrylate	SHOFU INC. Japan	092181
Filtek Supreme Flowable	A low viscosity, visible light curing flowable resin composite.	**Resin matrix**: Bis-GMA, Procrylat, and TEGDMA **Filler Content**: 20 nm silica fillers, 75 nm silica fillers, 0.1 to 5.0 μm ytterbium trifluoride **Filler loading**: (65 wt%, 46 vol%)	3 M Oral Care, USA	9,222,444
Filtek Z250 XT	A visible light curing nanohybrid resin composite	**Resin matrix**: Bis-GMA, UDMA, Bis-EMA and TEDGMA **Fillers**: Micro-sized or less surface-modified zirconia/silica particles. Nano-sized surface modified silica particles (82% by wt, 68 vol%).		NE57136

*Bis-GMA* Bis phenol glycidyl dimethacrylate, *TEGDMA* Triethylene glycol dimethacrylate, *UDMA* Urethanedimethacrylate, *Bis-EMA* bisphenol Aethoxylated methacrylate, *HEMA* 2-hydroxyethyl methacrylate, *MDP* Methacryloyloxydecyl dihydrogen phosphate

### Outcome assessment

Modified US Public Health Service (USPHS) criteria were selected for the clinical evaluation of the restorations [29]. The primary outcome was marginal adaptation, whereas secondary outcomes were gross fracture, marginal discoloration, color match and recurrent caries. The outcomes were assessed and documented by two experienced examiners at baseline (1 week after restoration placement), after three, six, 12 and 18 months in the evaluation charts. The scoring was: Alpha (clinically excellent), Bravo (clinically acceptable) and Charlie (clinically unsatisfactory). Calibration of the assessors was done to enhance the inter and intra-examiner reliability by reviewing 20 photographs representing different scores for all the evaluated criteria to achieve a kappa value of at least 90% for both inter- and intra-examiner agreement on each criterion.

The clinical assessment was conducted in a dental chair under dental operating light of 5000k and 25,000 lx, and the lighting parameters were the same at every recall visit to ensure the standardization and accuracy of the recorded data. Visual and tactile examinations were conducted using a dental mirror and a ballpoint probe with the aid of dental 3.5x magnification loupes to optimize the assessment. Photo documentation was done preoperatively and at every recall visit, including the restored tooth, the adjacent teeth and the surrounding tissues.

The participants were re-evaluated regarding the eligibility criteria at every recall visit particularly their oral hygiene to ensure their compliance with the given instructions. The restorations were cleaned and polished before the assessment with polishing brushes and prophylactic paste, then they were adequately rinsed and dried. During the assessment, the field was isolated with cotton rolls. Tactile examination was conducted by moving the tip of the ballpoint probe across the restoration margins in both directions to detect any marginal discrepancy. Color match was assessed by detecting any visual color or translucency difference from the surrounding enamel or the adjacent teeth.

### Statistical analysis

The data were analyzed using Medcalc software, version 22 for Windows (MedCalc Software Ltd, Ostend, Belgium). The frequencies and percentages were used to describe the categorical data. The chi-Square test was used for intergroup comparisons, with a statistical significance level set at *P* ≤ 0.016 after Bonferroni correction. The Cochran’s Q test was used for comparisons within each group, with a statistical significance level set at *P* ≤ 0.005 after Bonferroni correction. The clinical significance was evaluated on the basis of relative risk. The confidence level was established at 95% with a statistical power of 80%, and all tests were two-tailed.

## Results

**Fig. 2:**
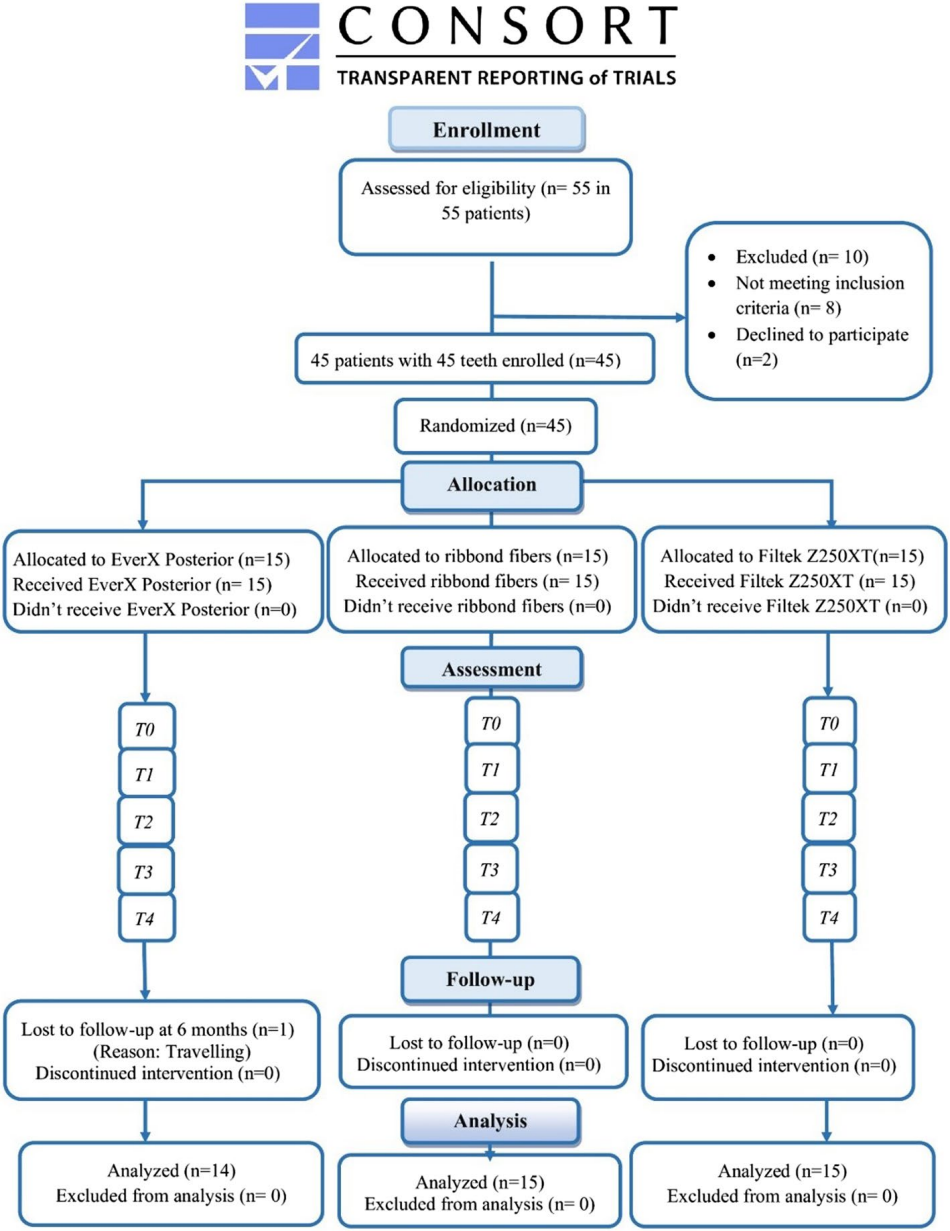
Participants flow diagram

After 18 months 44 participants completed the follow-up, with a 97.7% retention rate. Only one restoration was lost to follow-up at the 6-months examination. The study followed CONSORT 2010 flow reporting guidelines (Fig. 2). A total of 17 males (37.8%) and 28 females (62.2%) participated in this clinical trial. The mean age of the participants in this trial was 32.3 ± 7.3 years. The statistical analysis of both gender and age distribution revealed no significant differences (*P* = 0.9098, 0.950). The analysis of teeth distribution in the upper and lower arches revealed 23 maxillary premolars, 7 maxillary molars, 5 mandibular premolars, and 10 mandibular molars in the current study (*P* = 0.7015).

**Fig. 3:**
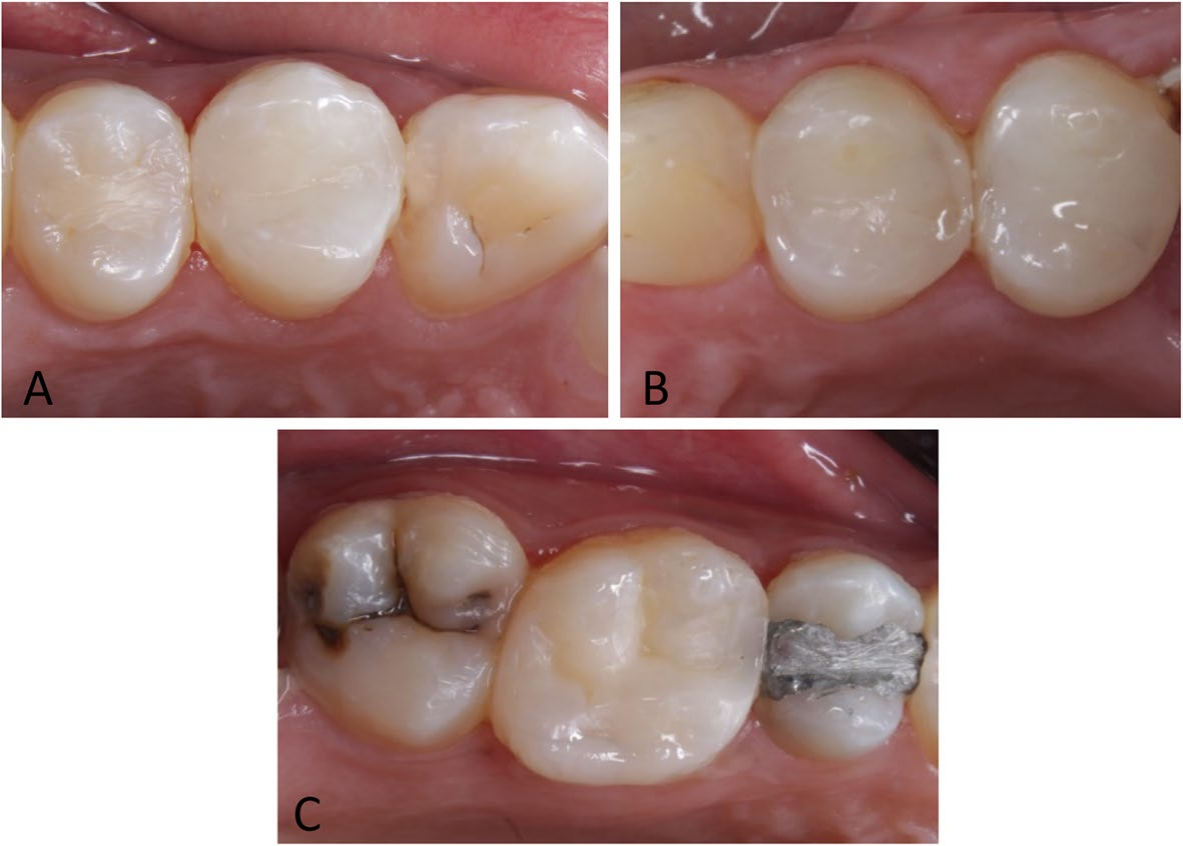
Representative cases of alpha scores for all evaluated criteria in different groups after 18 months’ follow-up. **A **Group 1 (tooth #24), **B **Group 2 (tooth #14),** C **Group 3 (tooth #26)

Comparisons among the three groups revealed no statistically significant difference in the primary outcome of marginal adaptation; After 18 months of follow up, only one restoration scored Bravo in both the short glass and polyethylene fiber reinforced composite groups as the probe caught during assessment and a gap was visible but there was no exposed dentin in comparison to three restorations in the control group. The analysis of the secondary outcomes revealed no statistically significant difference between all the groups except for color match; four restorations of the short fiber reinforced composite scored bravo from the baseline, recording statistically significant differences across all follow-up periods (*P* < 0.016) as there was a clinically acceptable deviation in translucency between the tooth and the restoration. In terms of marginal discoloration, all the restorations in the polyethylene fiber group scored Alpha after 18 months, whereas two restorations in the short fiber group scored Bravo and three restorations in the nanohybrid group scored Bravo indicating superficial marginal discoloration. All of the restorations in all the groups scored alpha in terms of both gross fracture and recurrent caries. For the comparisons within each group, there was no statistically significant difference between follow-up intervals regarding all criteria. A detailed analysis of the results for all the groups is presented in Table 3. Representative photographs at the end of 18 months are presented in Fig. 3.

**Table 3:** Frequency (n), percentage (%) for all the USPHS criteria

	Follow-up	Short glass fiber reinforced			Polyethylene fiber			Nanohybrid resin composite			*P* value
		A	B	C	A	B	C	A	B	C	
**Marginal Adaptation**	Baseline	15 (100%)	0 (0%)	0 (0%)	15 (100%)	0 (0%)	0 (0%)	15 (100%)	0 (0%)	0 (0%)	1.0000 (NS)
	3 months	15 (100%)	0 (0%)	0 (0%)	15 (100%)	0 (0%)	0 (0%)	15 (100%)	0 (0%)	0 (0%)	1.0000 (NS)
	6 months	15 (100%)	0 (0%)	0 (0%)	15 (100%)	0 (0%)	0 (0%)	13(86.7%)	2(13.3%)	0 (0%)	0.1233 (NS)
	12 months	14 (100%)	0 (0%)	0 (0%)	15 (100%)	0 (0%)	0 (0%)	13(86.7%)	2(13.3%)	0 (0%)	0.1319 (NS)
	18 months	13(92.9%)	1(7.1%)	0 (0%)	14(93.3%)	1(6.7%)	0 (0%)	12 (80%)	3 (20%)	0 (0%)	0.4302 (NS)
	*P* value	0.406 (NS)			0.406 (NS)			0.061 (NS)			
**Color Match**	Baseline	10(66.7%)	5(33.3%)	0 (0%)	15 (100%)	0 (0%)	0 (0%)	15 (100%)	0 (0%)	0 (0%)	0.0036*
	3 months	10(66.7%)	5(33.3%)	0 (0%)	15 (100%)	0 (0%)	0 (0%)	15 (100%)	0 (0%)	0 (0%)	0.0036*
	6 months	10(66.7%)	5(33.3%)	0 (0%)	15 (100%)	0 (0%)	0 (0%)	15 (100%)	0 (0%)	0 (0%)	0.0036*
	12 months	10(71.4%)	4(28.6%)	0 (0%)	15 (100%)	0 (0%)	0 (0%)	15 (100%)	0 (0%)	0 (0%)	0.0090*
	18 months	10(71.4%)	4(28.6%)	0 (0%)	15 (100%)	0 (0%)	0 (0%)	15 (100%)	0 (0%)	0 (0%)	0.0090*
	*P* value	1.0000 (NS)			1.0000 (NS)			1.0000 (NS)			
**Marginal Discoloration**	Baseline	15 (100%)	0 (0%)	0 (0%)	15 (100%)	0 (0%)	0 (0%)	15 (100%)	0 (0%)	0 (0%)	1.0000 (NS)
	3 months	15 (100%)	0 (0%)	0 (0%)	15 (100%)	0 (0%)	0 (0%)	15 (100%)	0 (0%)	0 (0%)	1.0000 (NS)
	6 months	14(93.3%)	1(6.7%)	0 (0%)	15 (100%)	0 (0%)	0 (0%)	14(93.3%)	1(6.7%)	0 (0%)	0.5926 (NS)
	12 months	13(92.9%)	1(7.1%)	0 (0%)	15 (100%)	0 (0%)	0 (0%)	13(86.7%)	2(13.3%)	0 (0%)	0.3496 (NS)
	18 months	12(85.7%)	2(14.3%)	0 (0%)	15 (100%)	0 (0%)	0 (0%)	12 (80%)	3 (20%)	0 (0%)	0.2067 (NS)
	*P* value	0.171 (NS)			1.0000 (NS)			0.075 (NS)			
**Gross Fracture**	Baseline	15 (100%)	0 (0%)	0 (0%)	15 (100%)	0 (0%)	0 (0%)	15 (100%)	0 (0%)	0 (0%)	1.0000 (NS)
	3 months	15 (100%)	0 (0%)	0 (0%)	15 (100%)	0 (0%)	0 (0%)	15 (100%)	0 (0%)	0 (0%)	1.0000 (NS)
	6 months	15 (100%)	0 (0%)	0 (0%)	15 (100%)	0 (0%)	0 (0%)	15 (100%)	0 (0%)	0 (0%)	1.0000 (NS)
	12 months	14 (100%)	0 (0%)	0 (0%)	15 (100%)	0 (0%)	0 (0%)	15 (100%)	0 (0%)	0 (0%)	0.9775 (NS)
	18 months	14 (100%)	0 (0%)	0 (0%)	15 (100%)	0 (0%)	0 (0%)	15 (100%)	0 (0%)	0 (0%)	0.9775 (NS)
	*P* value	0.406 (NS)			1.0000 (NS)			1.0000 (NS)			
**Recurrent Caries**	Baseline	15 (100%)	0 (0%)	0 (0%)	15 (100%)	0 (0%)	0 (0%)	15 (100%)	0 (0%)	0 (0%)	1.0000 (NS)
	3 months	15 (100%)	0 (0%)	0 (0%)	15 (100%)	0 (0%)	0 (0%)	15 (100%)	0 (0%)	0 (0%)	1.0000 (NS)
	6 months	15 (100%)	0 (0%)	0 (0%)	15 (100%)	0 (0%)	0 (0%)	15 (100%)	0 (0%)	0 (0%)	1.0000 (NS)
	12 months	14 (100%)	0 (0%)	0 (0%)	15 (100%)	0 (0%)	0 (0%)	15 (100%)	0 (0%)	0 (0%)	0.9775 (NS)
	18 months	14 (100%)	0 (0%)	0 (0%)	15 (100%)	0 (0%)	0 (0%)	15 (100%)	0 (0%)	0 (0%)	0.9775 (NS)
	*P* value	0.406(NS)			1.0000(NS)			1.0000(NS)			

*NS* Non-significant

*Statistically significant

## Discussion

The fiber reinforcement of resin composite is one of the enhancements where the filler system is enhanced with fibers either externally or internally to resist crack propagation. The external reinforcement includes polyethylene fibers, whereas the internal reinforcement includes short glass fiber reinforced resin composites that contain short discontinuous glass fiber within the filler system [27]. (Ever X Posterior) consists of a resin matrix, short E-glass fibers arranged randomly within the matrix, and inorganic fillers [30]. The fibers act as strengthening media mimicking the fibrous nature of dentin (collagen fibers embedded in the matrix) [31]. Furthermore, fiber reinforced resin composite (Ever X Posterior) has the ability to absorb stresses and disperse energy in a manner comparable to that of dentin, hence enhancing mechanical performance [32].

UHMWPE fiber reinforcing ribbon systems have been implemented to improve the toughness of resin composites, hence enhancing their durability in high stress bearing areas [33]. Polyethylene fibers were wetted first in a plain unfilled resin liquid as recommended by the manufacturer, to increase their wettability, enhance their impregnation within the resin composite and improve their clinical efficiency [34]. Polyethylene fibers were placed horizontally on both the pulpal and gingival floors. In addition, fibers were placed circumferentially lining the axial walls ensuring the coverage of a larger surface area by fibers, which improved the load bearing capacity and dissipation of forces equally over the large surface area and relaxed the polymerization shrinkage stresses [33].

In the present study. For enhanced restoration outcomes, the addition of a surface layer of nanohybrid composite over a fiber-reinforced composite is recommended by the manufacturer [27]. By combining the strength and support of fiber-reinforced composites with the aesthetic and surface properties of the nanohybrid composite, the restorations will be functionally robust and visually appealing thus improving the clinical success rates. The bilayered configuration of this restoration closely resembles that of a natural tooth, making it a biomimetic restoration [35].

The modified United States Public Health Service (USPHS) criteria were used for the clinical evaluation because of their widespread use for assessment of dental restorations [36]. Therefore, they were chosen to increase the validity of this randomized clinical trial for inclusion and pooling in any future systematic review about fiber reinforced resin composites.

The current study was conducted on 45 participants, 44 of whom completed the follow-up resulting in 97.7% retention rate. After 18 months, one patient dropped out at the 6-months examination follow up because of travel outside the country. Furthermore, gender, age, and teeth distribution did not significantly affect the clinical performance of either fiber reinforced or nanohybrid resin composite. The absence of statistically significant differences in the clinical assessment criteria between molars and premolars indicates that these restorative materials perform consistently across different types of posterior teeth. These findings suggest that fiber-reinforced composites provide comparable functionality and durability outcomes in both molars and premolars. The uniform performance can be attributed to the material’s ability to reinforce the structural integrity of the teeth effectively, regardless of their position in the mouth. Furthermore, the results could be attributed to the same finishing and polishing procedures that ensured a durable surface finish and polishing and the surface layer was restored with nanohybrid resin composite in all the groups.

Regarding the outcomes of marginal adaptation and discoloration, in all the intergroup and intragroup comparisons, no statistically significant differences observed across the groups in any of the follow-up periods. Marginal integrity and discoloration are usually linked to each other, which is a prime concern in the evaluation of dental restorative materials [37]. This could be attributed to the combined effects of the lower degree of polymerization shrinkage related to fiber optimization of the fiber reinforced resin composites and the oblique incremental technique used in addition to the formulation of the nanohybrid resin composite [38]. Polymerization shrinkage stresses depend on different factors, such as the monomer species, initiator type and concentration, filler characteristics, degree of conversion and elastic modulus [39]. The use of nano-sized fillers in nanohybrid resin composites allows for improved filler loading, thereby enhancing the mechanical properties. Additionally, to reduce polymerization shrinkage, a portion of the TEGDMA is replaced with PEGDMA in the Filtek Z250 resin technology [40].

The marginal adaptation results for the short fiber reinforced resin composite were consistent with those of previous studies [41–44]. The amount, aspect ratio, and fiber orientation are assumed to play a role in the stress-relaxation process. The aspect ratio is defined as the length of the fiber divided by its diameter. Because of the high aspect ratio of Ever X Posterior fibers, when the cavity is filled, the fibers intertwine. Furthermore, during the polymerization, shrinkage is reduced along the fibers‒matrix interface [44]. Therefore, the organic matrix between the fibers is able to contract but it retains its original horizontal dimensions [45]. Additionally, the existence of short glass fibers and polymethylmethacrylate (PMMA) in the matrix resulted in less volumetric shrinkage than that of conventional resin composites [26]. Furthermore, the translucent nature of the SFRC allows the penetration of light to a greater depth resulting in a high polymerization rate and an optimal marginal seal [46]. Salem et al. [38] reported no significant difference regarding marginal adaptation, according to their findings, the stresses at the tooth-restoration interface where the majority of the polymerization shrinkage stress was applied, were absorbed by the intewined short glass fibers.

Regarding polyethylene fibers, the results were in accordance with those of a study conducted by Özüdoğru and Tosun [28] who attributed the results to an elastic shock absorber created at the cavity base by a flowable composite and polyethylene fibers, which helps to reduce polymerization stresses and enhance marginal integrity. Fibers could effectively minimize microleakage around the restoration which could be attributed to their high elastic modulus and low flexural modulus which mitigate the stresses that develop at the dentin/resin interface in contrast to restorations without fibers [47].

In contrast to the results of the present study, Guney and Yazici [39] reported significant differences within all groups for marginal adaptation and discoloration regarding short fiber reinforced resin composite restorations. They attributed these differences to the formulation of the tested resin composites, such as filler content, organic matrix specifications and polymerization shrinkage, which are the primary contributing factors affecting the durability of composite restorations. Disagreements in the results between the present study and the aforementioned study can be attributed to the different follow up periods and the bonding agents used.

When considering gross fracture, all restorations in the three groups had an alpha score at the end of the18 month period. The results could be attributed to effective stress transfer between the fibers and the cured resin matrix resulting in favourable strengthening effects in multiple directions [48]. The fibers of Ribbond are arranged in a tightly woven framework with nodal junctions, thus individual fibers act as crack stoppers [5]. Furthermore, in the current study the polyethylene fibers were positioned directly against the walls of the cavity, mimicking the dentino-enamel complex. This allows the tooth substrate and restorative composite to work together harmoniously under load [16]. These results were consistent with those of Agrawal et al. [33] who reported that applying polyethylene fibers to the pulpal and gingival floor can enhance the ability of these fibers to withstand and distribute stresses evenly across the extensive surface area.

In contrast, in a clinical trial, Van Dijken and Sunnegårdh-Grönberg [49] compared two types of fiber reinforced resin composites. The failure was attributed to two factors. First, the FRC materials were packed incrementally to fill the entire cavity, in contrast to the FRC that is currently available on the dental market, which is recommended to be capped by an occlusal and a proximal (in the case of class II cavities) particulate-filled composite layer. Second, the FRC used in that study had a fiber length below the critical fiber length that is recommended for adequate reinforcement of the resin matrix. The critical length is defined as the minimum dimension of aligned fibers fulfilling adequate stress transfer between the fibers and the cured composite matrix [50].

Regarding the results of the color match, four short fiber reinforced composite restorations scored bravo showing statistically significant difference. The manufacturer’s instructions advocate the application of a 1–2 mm occlusal layer of conventional composite for EverX Posterior restorations to obtain the best possible aesthetics [26]. However, the present study revealed that even with this thickness of covering nanohybrid composite, there was a minor deviation in translucency which could be attributed to the unique translucency of EverX Posterior. The addition of glass fibers to resin composites could alter the optical properties and the translucency of composite restorations as shown in in-vitro studies [51, 52]. Furthermore, the visual shade assessment method employed in the present study could be subjective and affected by parameters such as lighting conditions, eye fatigue and color perception. These results were in agreement with those of Elaziz et al. [27] who attributed their results to the ineffectiveness of the remaining occlusal layer of hybrid resin composite used in their trial to mask the discolored dentin left at the cavity floor. Therefore, to achieve optimal aesthetic outcomes, it is advisable to apply an underlying opaquer below (Ever X Posterior) or increase the thickness of the covering material by more than 2 mm.

For the results of secondary caries, favourable results were attributed to factors such as the brief duration of the observation period, proper oral and dental hygiene maintenance, precise placement technique of the clinician and improvements in the adhesive strategies and restorative materials. Patient related factors are the primary factors that are crucial in determining the formation of secondary caries regardless of the status of marginal integrity [28].

On the basis of these previous findings, the proposed null hypothesis was accepted as fiber reinforced resin composite restorations with either short glass or polyethylene fibers showed similar clinical performance as conventional nanohybrid resin composite restorations after 18 months of follow up. Together, the dentist and patient should consider a number of factors when choosing between these materials. Polyethylene fibers need more time and experience for precise application, resulting in cost ineffectiveness. Both short fiber reinforced and conventional resin composites are easier to apply and more time saving.

One of the limitations of this study was the short follow-up period, but additional follow-up appointments are planned. Moreover, the categories of the modified USPHS might not accurately represent the clinical success of the restorations. It is advised to make use of both the FDI and modified USPHS methods is advised to guarantee the early identification of signs that restorations are deteriorating. Furthermore, although patients with evidence of parafunctional habits were excluded, analysis of patients’ occlusion wasn’t a point of focus. Finally, operator blinding was inapplicable due to the nature of the restorative materials used in the study.

## Conclusions

Under the parameters of this study and based on the results, it was concluded that:

1. Class II direct fiber-reinforced resin composite restorations can offer acceptable performance similar to the nanohybrid resin composite through 18-month of clinical service.
2. Practical considerations like application complexity and cost may influence material selection.
3. Short glass fiber-reinforced restorations exhibited minor deviations in color match, emphasizing the need for careful aesthetic considerations.

### Recommendations

1. To substantiate the current results, a longer follow-up period is highly recommended to observe all relevant effects and differences.
2. Further randomized clinical trials evaluating the general performance of fiber reinforced resin composite restorations in various clinical situations are recommended.
3. Utilization of the FDI criteria in conjunction with the modified USPHS criteria to compare the results of both restoration assessment methods.

## Data Availability

The datasets used and/or analyzd during the current study are available from the corresponding author on reasonable request.

## Abbreviations

FRCs: Fiber-reinforced composites
MOD: Mesio-occlusal-distal
LED: Light-emitting diode
USPHS: Modified US Public Health Service
DEJ: Dentin-enamel junction
SFRC: Short fiber reinforced resin composite
